# Clinical Utility of Boston-CTS and Six-Item CTS Questionnaires in Carpal Tunnel Syndrome Associated with Diabetic Polyneuropathy

**DOI:** 10.3390/diagnostics13010004

**Published:** 2022-12-20

**Authors:** Nicu Cătălin Drăghici, Daniel-Corneliu Leucuța, Dana Mihaela Ciobanu, Adina Dora Stan, Tudor Dimitrie Lupescu, Dafin Fior Mureșanu

**Affiliations:** 1 IMOGEN Institute, Centre of Advanced Research Studies, 400012 Cluj-Napoca, Romania; 2 RoNeuro Institute, Centre for Neurological Research and Diagnostic, 400364 Cluj-Napoca, Romania; 3 Department of Clinical Neurosciences, Iuliu Hațieganu University of Medicine and Pharmacy, 400012 Cluj-Napoca, Romania; 4 Department of Medical Informatics and Biostatistics, Iuliu Hațieganu University of Medicine and Pharmacy, 400349 Cluj-Napoca, Romania; 5 Department Diabetes and Nutrition Diseases, Iuliu Hațieganu University of Medicine and Pharmacy, 400006 Cluj-Napoca, Romania

**Keywords:** carpal tunnel syndrome, diabetic polyneuropathy, nerve conduction studies, Boston Carpal Tunnel Syndrome Questionnaire, six-item Carpal Tunnel Syndrome symptoms scale

## Abstract

Diabetic polyneuropathy (DPN) is the most frequent complication of diabetes. Carpal tunnel syndrome (CTS), one of the most common neuropathies, is a chronic compression of the median nerve at the wrist. In our prospective cross-sectional study, we enrolled patients with type 2 diabetes presenting with signs and symptoms suggestive of DPN (*n* = 53). We aimed to compare two clinical scales: the Boston Carpal Tunnel Syndrome Questionnaire (BCTQ) and the six-item CTS symptoms scale (CTS-6), with nerve conduction studies (NCS) for detecting CTS in patients with DPN. Carpal tunnel syndrome and DPN were clinically evaluated, and the diagnosis was confirmed by NCS. Depending on the NCS parameters, the study group was divided into patients with and without DPN. For each group, we selected patients with CTS confirmed through NCS, and the results were compared with the BCTQ and CTS-6 scales. The clinical evaluation of CTS performed through BCTQ and CTS-6 was statistically significantly different between patients with and without CTS. When comparing the BCTQ questionnaire with the NCS tests, we found area under the curve (AUC) = 0.76 (95% CI 0.65–0.86) in patients with neuropathy and AUC = 0.72 (95% CI 0.55–0.88) in patients without neuropathy. At the same time, the AUC values of the CTS-6 scale were 0.76 (95% CI 0.61–0.88) in patients with neuropathy and 0.70 (95% CI 0.51–0.86) in patients without neuropathy. Using multiple logistic regression, we demonstrated that DPN increased the chances of detecting CTS using the two questionnaires. The Boston Carpal Tunnel Syndrome and CTS-6 questionnaires can be used in the diagnosis of CTS in diabetic patients with and without DPN but with moderate AUC. The presence of DPN increased the chances of detecting CTS using the BCTQ questionnaire and the CTS-6 scale.

## 1. Introduction

The prevalence of diabetes mellitus is increasing worldwide and constitutes a real public health problem, affecting around 9.3% of the global population and expected to rise to 10.3% by 2045 [1]. Diabetic polyneuropathy (DPN) is the most frequent long-term complication of diabetes, and the duration of diabetes associated with poor glycemic control represents the main risk factor [2]. Moreover, several factors, such as obesity and metabolic syndrome, are linked to the development of DPN [3]. The pathophysiology of neuropathy has not been fully elucidated, but the leading cause of peripheral nerve damage appears to be apoptosis caused by glucose toxicity [4].

Carpal tunnel syndrome (CTS) is one of the most common neuropathies and is characterized by chronic compression of the median nerve at the level of the flexor retinaculum. It is commonly encountered in patients with diabetes and hypothyroidism, obese women, and repetitive action workers [5]. Several biochemical and structural factors contribute to the susceptibility of peripheral nerve compression in diabetes [6], but at the same time, some authors consider that the relationship between diabetes and CTS occurrence remains unclear [7,8].

Several investigations, such as ultrasonography and nerve conduction studies (NCS), have been intensively studied in the last decade, showing good accuracy in diagnosing CTS in the general population as well as in patients with diabetes [9,10,11]. At the same time, clinical scales have been developed and are frequently used for the diagnosis of CTS in the general population. The Boston Carpal Tunnel Syndrome Questionnaire (BCTQ) is a self-administered clinical scale developed by Levine et al. and used to assess the function and symptom severity of CTS [12]. A few years later, using a modern measurement methodology, Atrosi et al. proposed a six-item CTS symptoms scale (CTS-6) composed of a short set of questions for assessing median nerve compression [13]. However, the aforementioned scales have been evaluated in the general population, but we have no references for patients with diabetes, in particular in subjects with length-dependent polyneuropathy associated with median nerve compression at the radio-carpal joint. The study’s aim was to evaluate the diagnostic accuracy of the BCTQ—symptom severity scale and the CTS-6 scale compared with the NCS tests for detecting the presence of CTS in patients with DPN.

## 2. Materials and Methods

### 2.1. Study Population

In this prospective cross-sectional study, we enrolled consecutively a total of 53 adult patients diagnosed with type 2 diabetes and signs and symptoms suggestive of DPN: (a) lower limb paresthesia and (b) abolished Achilles reflexes [14], presenting at the neurophysiology laboratory of the IMOGEN Institute, Cluj-Napoca, Romania. The presence of other causes of peripheral neuropathies, such as vitamin B12 and folic acid deficiency, chronic alcohol consumption, and malignancies, were considered exclusion criteria [15].

All investigations were conducted in accordance with the guidelines in The Declaration of Helsinki. The local Ethics Committee of the “Iuliu Haţieganu” University of Medicine and Pharmacy, Cluj-Napoca, Romania, approved the study protocol (approval number 26/9.02.2016). All participants provided written informed consent before undergoing an examination by medical staff.

Data regarding age, sex, body mass index, duration of diabetes, fasting blood glucose, and treatment with insulin were collected from the patient interviews, medical files, and physical examinations. All patients underwent NCS examination using a Natus CareFusion Viking machine. A neurophysiologist with four years of experience performed all the examinations, and the upper and lower limb temperature was maintained above 32 °C. In order to highlight the peripheral nervous system damage, we performed the nerve conduction velocities for sural nerves and left superficial peroneal, tibial, and fibular nerves. The DPN diagnosis was validated based on the clinical examination and was confirmed through NCS. In our study, a sural sensory nerve action potential amplitude (SNAP) < 20% of the normal value was considered pathological [16]. Moreover, we extended the NCS, and we performed needle electromyography (EMG) in the left vastus lateralis and tibialis anterior muscles to rule out radiculopathy or neuromuscular diseases [17].

### 2.2. Diabetic Polyneuropathy Evaluation

In order to quantify the severity of peripheral nerve damage, we calculated the sum of bilateral sural nerve sensory responses and divided patients into two groups according to this value. In the first group, we included patients whose SNAP amplitude sum was ≤15 µV. This group was represented by patients with DPN. The second category included patients with the sum of SNAP amplitude responses of the two sural > 15 µV. This group was represented by patients without polyneuropathy. The sural nerve was recorded antidromically according to the standard electroneurophysiological techniques proposed by E. Fournier [16]. The ENG parameters were recorded using Kandle adhesive skin electrodes, and the nerve stimulation was performed using the bar electrode, following the standard of intensity and duration techniques. The active electrode (A1) was positioned below the external malleolus, and the reference electrode (A2) was positioned two centimeters distal to this level. The nerve stimulation was performed on the posterior side of the calf, using a biphasic current of about 30 mA, with a duration of 0.1–0.2 ms. To decrease artifacts and to ensure optimal stimulation, we used an electrode conductive gel. In addition, the patients were asked to confirm the sensation of irradiation on the lateral side of the foot. Moreover, for each subject enrolled in the study, we applied the Toronto scale, which includes the sum of subjective sensitive symptoms (pain, numbness, paresthesia, muscle weakness, ataxia), the tendon reflex score, and sensitivity testing [18].

### 2.3. Carpal Tunnel Syndrome Evaluation

The patients included in the study received NCS in the bilateral upper limb. Thus, for a possible median nerve compression neuropathy, we used two techniques with high sensitivity and specificity: (1) the difference between the median motor distal latency and the ulnar motor distal latency (lumbricals II—interossei II), and (2) the difference between the median and the ulnar sensory distal latencies (digit IV) [19]. The NCS was performed with Kandle adhesive electrodes. The active electrode (A1) was positioned in the palm, in the space between the second and the third metacarpal, above the lumbricals II and the interossei II muscles. The nerve was stimulated at classical points, about 10 cm from the active electrode. The active electrode was positioned at a common point, at the proximal interphalangeal joints of finger IV, above 14 cm from the stimulation electrode in order to calculate the sensory distal latencies difference between the median and the ulnar nerve [20]. In our study, NCS was considered the gold standard, and a difference between the median and the ulnar nerve sensory distal latencies > 0.5 ms or a difference between the median and the ulnar motor distal latency > 0.5 ms was diagnostic for CTS [19,20].

The clinical diagnosis of CTS was established by the presence of numbness or tingling in at least two of the digits I, II, III, and ½ of IV persisting for at least one month [19]. In addition, we applied the BCTQ—symptom severity score and calculated the CTS-6 score for all the patients included in the study. The two questionnaires and the NCS examination were performed on the same day.

### 2.4. Statistical Analyses

Qualitative data were described through absolute and relative frequencies. Normally distributed quantitative data were described by mean and standard deviation, and those that did not follow a normal distribution were described by median and quartiles. Comparisons between two independent groups were made for qualitative data through the Hi2 test or Fisher’s exact test. For quantitative data, if the values were normally distributed, statistical tests were performed through the Student test for independent samples and through the Mann–Whitney U test if the values were not normally distributed.

A multiple logistic regression model was performed with CTS as the dependent variable and the BCTQ scale and polyneuropathy (yes vs. no) as independent variables. Model fit was checked through the Hosmer–Lemeshow test, and multicollinearity was checked through the variance inflation factor. The assumption of log-linearity for the quantitative variable was checked through a general additive model using a spline function. The results were presented through the odds ratio with associated confidence interval and statistical significance value. Receiver operating characteristic curves were made to assess the diagnostic value for different variables in classifying the presence of CTS. We calculated the area under the curve (AUC) with the associated confidence interval for each such curve using a bootstrapping method.

For all tests, the value 0.05 was used as the statistical significance threshold, and bilateral *p*-values were calculated. Statistical analysis was performed in the R (Vienna) statistics and graphics environment, version 4.0.2.

## 3. Results

In our study, we included 53 patients with diabetes presenting with signs and symptoms suggestive of DPN. After performing NCS, the study subjects were divided into two groups, and 35 patients were diagnosed with DPN through NCS. The demographic and clinical data of the subjects with and without DPN are shown in Table 1.

In addition, the two groups of patients (with/without DPN) were divided into two subgroups according to the presence or absence of CTS confirmed through NCS. More details of the results can be consulted in Table 2. Moreover, we calculated the AUC of the BCTQ and CTS-6 scores according to the NCS values diagnostic for CTS for all patients. In Table 2, for the BCTQ and CTS-6 questionnaires, the results show a statistically significant difference between the two subgroups of patients—with and without NCS-confirmed CTS.

**Figure 1:**
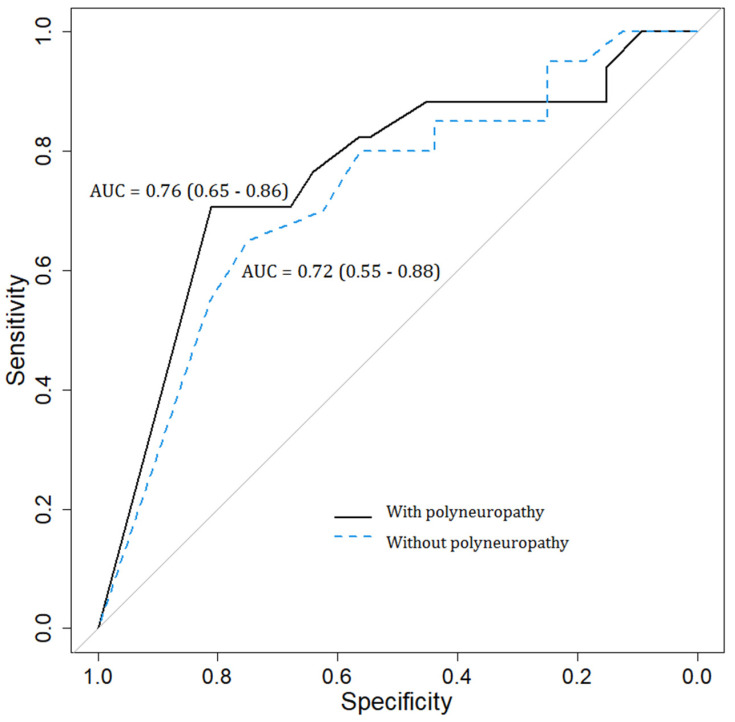
Area under the curve values of the BCTQ questionnaire according to nerve conduction studies in patients with and without diabetic polyneuropathy.

**Figure 2:**
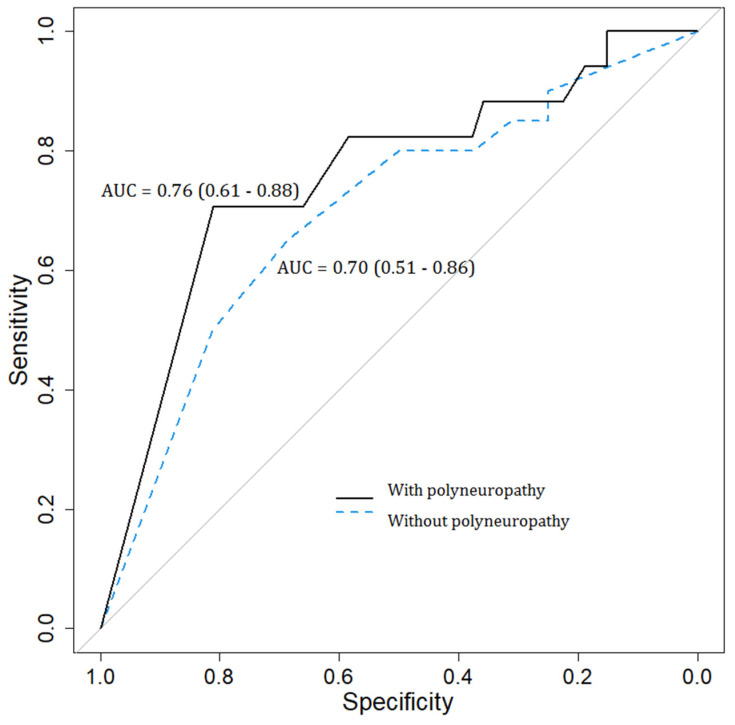
Area under the curve values of the CTS-6 questionnaire according to nerve conduction studies in subjects with and without diabetic polyneuropathy.

The AUC value of the BCTQ and CTS-6, according to the NCS parameters in patients with and without DPN, can be consulted in Figure 1 and Figure 2. Therefore, in Figure 1, the BCTQ questionnaire AUC values for patients with polyneuropathy were relatively low, with an AUC = 0.76 (0.65–0.86), compared to the reference examination represented by NCS. Similarly, the AUC of the BCTQ questionnaire in the detection of CTS in subjects without polyneuropathy was diminished, with an AUC value = 0.72 (0.55–0.88). Moreover, in Figure 2, the AUC values of the CTS-6 scale in comparison with the NCS tests were reduced in both groups of patients, with AUC = 0.76 (0.61–0.88) in patients with polyneuropathy and AUC = 0.70 (0.51–0.86) in patients without polyneuropathy.

We used a multiple logistic regression, which attempted to predict CTS confirmed by NCS, based on two independent variables: the BCTQ score and the presence of DPN (confirmed NCS), respectively, the CTS-6 score and the presence of DPN (Table 3). We observed that the odds of diagnosing CTS through NCS were 3.59 times higher in subjects who had DPN compared to those without polyneuropathy, the association being statistically significant. Likewise, for each additional unit of BCTQ score, the odds of a diagnosis of CTS through NCS were 3.65 times higher, the association being statistically significant. Moreover, for the CTS-6 model, the odds of confirming the diagnosis of CTS through NCS were 2.53 times higher in patients with DPN compared to patients without polyneuropathy, and for each additional unit of the CTS-6 score, the odds of CTS diagnosis by NCS were 3.09 times higher. In other words, it seems the presence of DPN increased the chances of detecting CTS using the BCTQ questionnaire and the CTS-6 score.

## 4. Discussion

Our study managed to evaluate the diagnostic accuracy of the BCTQ—symptom severity scale and the CTS-6 scale compared with NCS tests in patients with DPN, finding moderate diagnostic accuracies, as reflected by AUCs. Furthermore, the BCTQ questionnaire and the CTS-6 scale had a higher likelihood of identifying CTS when polyneuropathy was present.

Diabetic polyneuropathy is a multifactorial disease resulting from a combination of factors, of which the duration of diabetes and high blood glucose levels seem to be the main conditions that induce neuropathy [21]. In addition to this incompletely elucidated pathophysiology, there are other elements represented by factors included in metabolic syndrome, smoking, cardiovascular disease, or hypertension [22].

From the early stage of the results of our study, when analyzing the distribution of patients with and without DPN, we noticed that the number of subjects treated with insulin was higher in the group of patients with DPN. These data can be explained by the relation of insulin requirement versus diabetes duration, but also in the context of insulin requirement versus fasting blood glucose value. Thus, recent studies demonstrated that a longer duration of diabetes involves complex therapy, including insulin [23]. However, even if the diabetes duration is longer in patients treated with insulin, this does not fully explain the occurrence of DPN, which can be encountered in 11–25% as early as the pre-diabetes stage [24]. Moreover, fasting blood glucose and HbA1c were significantly higher in patients with DPN, which requires a variety of clinical strategies to reduce hyperglycemia, including insulin therapy [25,26]. At the same time, a study evaluating the relationship between insulin and DPN highlighted the neurotrophic potential of the hormone but also discussed the loss of normal neuronal insulin signaling in diabetes, which is, in fact, one of the main factors playing a role in DPN dysfunction and neuropathy symptoms [27]. When achieving rapid glycemic control, one study showed that insulin treatment might induce neuropathy (insulin neuritis), characterized by acute and severe pain, peripheral nerve degeneration, and autonomic dysfunction [28].

Moreover, in the first part of the results, we noticed that the CTS symptoms duration was longer in patients without polyneuropathy. This remark is confirmed by recent studies attesting that the incidence of CTS was higher in patients without DPN, which may suggest different mechanisms in the two pathologies [29]. However, if DPN was present, the median nerve was more susceptible to pressure in the carpal tunnel. Therefore, another possibility is that polyneuropathy may lead to the accentuation of CTS symptoms, thus determining a faster diagnosis and prompt treatment that induces a shorter duration of CTS symptoms [30].

Nevertheless, symptoms such as numbness and tingling in the upper limbs are part of the spectrum of DPN symptoms but may also be due to the compression of the median nerve at the carpal level, which may lead to diagnostic confusion [31,32]. In these circumstances, the Toronto Diabetic Neuropathy Expert Group described the variability of the clinical examination in subjects with DPN and demonstrated that compared to NCS, which has a low coefficient of variability, the clinical examination is inconsistent and sometimes leads to overdiagnosis [33].

In our case, using NCS as the reference standard, the BCTQ and the CTS-6 scores were statistically significantly different in the group of patients with CTS versus the group without CTS, demonstrating that both questionnaires can be used for CTS assessment in diabetic patients. At the same time, in subjects with DPN, the presence of neuropathy increased the odds of diagnosing CTS using the BCTQ questionnaire and the CTS-6 score. In addition, when we studied the AUC values of the BCTQ questionnaire and the CTS-6 score according to NCS measurements, we noticed a moderate value in both groups of patients, with and without DPN. Conversely, a recent study reported that electrodiagnostic testing and median nerve ultrasound could not distinguish between patients with and without symptoms of CTS. Hence, the authors concluded that the diagnosis of CTS in patients with diabetes should rely on clinical symptoms and signs [34], although a recent expert consensus reported that combining electrodiagnostic methods with ultrasonography increases the chances of diagnosing CTS [35]. The explanation for the difference between the two studies might be related to the fact that we used a method with high sensitivity and specificity for diagnosing CTS [19] compared to the method described in the study of Heiling et al. [34].

At the same time, as we already mentioned in the introductory part of the study, diabetes duration is a risk factor for DPN development. This note is in antithesis to our results, in which diabetes duration and the duration of neuropathy symptoms were not correlated with the presence of DPN confirmed through NCS, the *p*-value in our case being close to the statistical threshold. The explanation may be the occurrence of small fiber neuropathy, present since the early stages of diabetes, clinically described by the patients through a series of sensory symptoms, the most important of which is neuropathic pain [22]. It is worth mentioning that small fiber neuropathy is not detected through NCS tests assessing thick fibers; other instruments are needed to detect it, the most relevant of which is a skin biopsy [36]. Furthermore, in patients with DPN, the results showed that the Toronto score, which quantifies the severity of neuropathy, had a higher value in patients with CTS. This can be explained by the combination of symptoms of the two diseases and the higher prevalence of CTS in patients with DPN [37].

This research presented a series of limitations, such as the small number of patients included in the study and the examiner not being blinded for NCS testing, and the outcome of clinical scales. However, the study has a strong clinical relevance. This research demonstrates the utility of BCTQ and CTS-6 in the assessment of median nerve compression at the carpal level. Thus, any medical practitioner, regardless of experience, can use these scales in the CTS screening of patients with diabetes. Nonetheless, performing NCS requires time and special equipment, and it is usually performed by a physician with experience in neurophysiology.

## 5. Conclusions

The major finding of our study is that the two clinical scales BCTQ and CTS-6 could be used in the diagnosis of carpal tunnel syndrome in patients with diabetes. Compared to nerve conduction studies, both the BCTQ and CTS-6 questionnaires increased the chances of detecting carpal tunnel syndrome in patients with diabetic polyneuropathy.

## Figures and Tables

**Table 1:** Demographic data and clinical distribution of patients with and without diabetic polyneuropathy.

Variable	Polyneuropathy (Yes) (*n* = 35 Patients)	Polyneuropathy (No) (*n* = 18 Patients)	*p*-Value
Age (years), average (DS)	62.91 (6.35)	61.33 (5.89)	0.388 *
Gender (F), no (%)	19 (54.29)	11 (61.11)	0.635 **
Patient with insulin (yes), no (%)	19 (54.29)	4 (22.22)	0.026 **
Glycaemia (mg/dL), average (DS)	131.94 (20.35)	122.67 (18.09)	0.111 *
Body mass index (kg/m^2^), average (DS)	32.47 (5.07)	30.25 (3.61)	0.108 *
Diabetes duration (months), median (IQR)	120 (90–180)	86 (36–144)	0.05 *
Insulin duration (months), median (IQR)	4 (0–45)	0 (0–0)	0.066 *
Polyneuropathy symptom duration (months), median (IQR)	36 (12–48)	12 (6–36)	0.137 *
Carpal tunnel syndrome symptom duration (months), median (IQR)	6 (0–24)	18 (0–36)	0.026 ^●^

The values are reported as an average (SD), respectively median (Quartile 1; Quartile 3), * Student test for independent samples, ** Hi^2^ test, **^●^** Mann–Whitney U test.

**Table 2:** Characteristics of patients with/without diabetic polyneuropathy and carpal tunnel syndrome confirmed using nerve conduction studies.

Parameters	Carpal Tunnel Syndrome Confirmed Using Nerve Conduction Studies		Difference (95% CI)	*p*-Value *
**Patients with polyneuropathy**	Yes (*n* = 53 wrist)	No (*n* = 17 wrist)		
BCTQ score, median (IQR)	1.55 (1.18–2.18)	1 (1–1.27)	0.55 (0.09–0.73)	0.001
CTS-6 score, median (IQR)	1.67 (1.17–2.33)	1 (1–1.33)	0.67 (0.17–0.67)	0.001
Toronto score, median (IQR)	7 (6–8)	5 (4–6)	2 (0–3)	0.016
Difference motor median vs. ulnar (LII vs. IOD II) (ms), median (IQR)	0.6 (0.4–1.1)	0.2 (0.1–0.3)	0.4 (0.3–0.7)	<0.001
Difference sensory median vs. ulnar (Digi IV) (ms), median (IQR)	1 (0.8–1.8)	0.3 (0.2–0.3)	0.7 (0.6–1.1)	<0.001
**Patients without polyneuropathy**	Yes (*n* = 16 wrist)	No (*n* = 20 wrist)		
BCTQ score, median (IQR)	1.36 (1.16–1.87)	1 (1–1.27)	0.36 (0–0.46)	0.019
CTS-6 score, median (IQR)	1.5 (1.17–2.04)	1.08 (1–1.3)	0.41 (0–0.67)	0.041
Toronto score, median (IQR)	2.5 (2–5)	5 (3–6)	2.5 (−3–0)	0.133
Difference motor median vs. ulnar (LII vs. IOD II) (ms), median (IQR)	0.7 (0.4–1.22)	0.2 (0.1–0.3)	0.5 (0.2–0.8)	<0.001
Difference sensory median vs. ulnar (Digi IV) (ms), median (IQR)	1.05 (0.78–1.55)	0.2 (0.1–0.3)	0.85 (0.6–1.2)	<0.001

BCTQ = Boston Carpal Tunnel Questionnaire, CTS 6 = six-item CTS symptoms scale, L II = lumbrical II muscle, IOD II = interossei II muscle, CI = confidence interval. The values are reported as median (Quartile 1; Quartile 3); * Mann–Whitney U test.

**Table 3:** Multiple logistic regression having CTS dependent variable predicted according to the Boston carpal tunnel questionnaire and the six-item CTS symptoms scale and diabetic polyneuropathy.

	OR Adjusted	(95% CI)	*p*-Value
BCTQ model			
BCTQ Score	3.65	(1.62–9.97)	0.004
Polyneuropathy (yes vs. no)	3.59	(1.47–9.04)	0.006
CTS-6 model			
CTS-6 score	3.09	(1.52–7.37)	0.005
Polyneuropathy (yes vs. no)	2.53	(1.35–4.88)	0.004

BCTQ score = Boston tunnel carpal questionnaire; CTS-6 = six-item CTS symptoms scale. OR—odds ratio; CI—confidence interval.

## Data Availability

The data presented in this study are available on request from the corresponding author. The data are not publicly available as written consent was not obtained from study participants for this.
